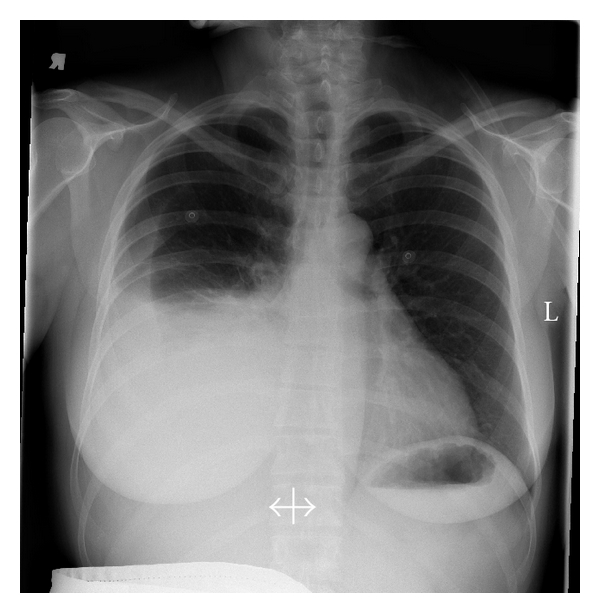# Symptomatic Isolated Pleural Effusion as an Atypical Presentation of Ovarian Hyperstimulation Syndrome

**DOI:** 10.1155/2011/967849

**Published:** 2011-08-07

**Authors:** Christine M. Mullin, M. Elizabeth Fino, Andrea Reh, Jamie A. Grifo, Frederick Licciardi

**Affiliations:** NYU Fertility Center, New York University, 660 First Avenue, 5th Floor, New York, NY 10016, USA

## Abstract

Ovarian hyperstimulation syndrome (OHSS) presents in ~33% of ovarian stimulation cycles with clinical manifestations varying from mild to severe. Its pathogenesis is unknown. Pleural effusion is reported in ~10% of severe OHSS cases and is usually associated with marked ascites. The isolated finding of pleural effusions without ascites, as the main presenting symptom of OHSS is not frequently reported and its pathogenesis is also unknown. We describe two unusual cases of OHSS where dyspnea secondary to unilateral pleural effusion was the only presenting symptom. By reporting our experience, we would like to heighten physicians' awareness in detecting these cases early, as it is our belief that the incidence of pleural effusion in the absence of most commonly recognized risk factors for OHSS may be underestimated and may significantly compromise the health of the patient if treatment is not initiated in a reasonable amount of time.

## 1. Background

Ovarian hyperstimulation syndrome (OHSS) presents in ~33% of ovarian stimulation cycles with clinical manifestations varying from mild to severe [1]. Its pathogenesis is unknown, but the main pathophysiology is an increase of vascular permeability and third spacing, leading to hemoconcentration and inadequate end organ perfusion. Severe OHSS symptoms include massive ovarian enlargement with multiple cysts, rapid weight gain, and third space sequestration. Although the incidence of severe OHSS may be underreported at 0.5–0.96% [2–4], it still can be life threatening as a result of its potential to progress to adult respiratory distress syndrome, renal failure, hepatic injury, hemostatic imbalances, and thromboembolic episodes. 

Pleural effusion is reported in ~10% of severe OHSS cases and is usually associated with marked ascites [5, 6]. When there is significant ascites, it has been proposed that diaphragmatic lymphatics provide a route for the transfer of the ascitic fluid into the right pleural space since the right side of the chest contains the thoracic duct, the main channel for lymphatic drainage from the lower parts of the body [7]. The isolated finding of pleural effusions without ascites, as the main presenting symptom of OHSS, is not frequently reported [6]. A Canadian study reported an incidence of 0.65% (5/771 patients stimulated with gonadotropins) with only one patient requiring a thoracentesis (1/771, 0.12%) [8]. The pathophysiology of isolated pleural effusion in OHSS cases is also unknown. One hypothesis is that the pleural effusion occurs as a result of mildly increased intra-abdominal pressure that drives ascitic fluid through weak diaphragmatic anatomical defects. When these defects rupture, the negative intrathoracic pressure preferentially allows for the ascites to permeate through these open channels and enter the pleural space. These defects are more commonly seen on the right side of the diaphragm [9] and have been observed via laparoscopy, open thoracotomy, and postmortem as multiple macroscopic defects of thin membranes covering the tendinous portion of the diaphragm [5, 10].

By reporting our experience, we would like to heighten physicians' awareness in detecting these cases early, as it is our belief that the incidence of pleural effusion in the absence of more commonly recognized risk factors for OHSS may be underestimated.

### 1.1. Case  1

A 25-year-old G_0_ woman with no significant past medical history or physical findings other than polycystic ovaries on ultrasound enrolled in our Ooctye Donor Program. Downregulation was achieved using OCPs and depot leuprolide acetate 3.75 intramuscularly on day 21. Ovarian stimulation was initiated with 225 IU of human menopausal gonadotropin (hMG) for 5 days. This dose was tapered as a result of the patient's ovarian response, measured by ultrasound and estradiol levels. She received a total of 1975 IU of hMG over 9 days and 500 *μ*g of r-hCG when two follicles reached a 17 mm diameter; her peak estradiol level was 3,731 pmol/L. A total of 44 oocytes were retrieved.

**Figure 1:**
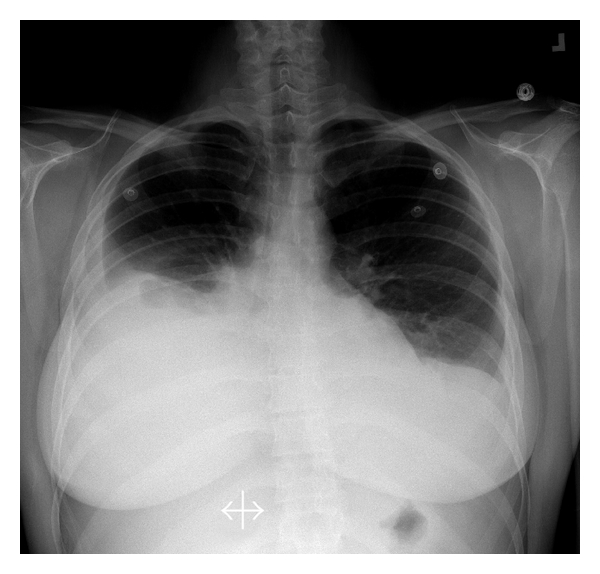


The patient initially presented 2 days after oocyte retrieval with complaints of dyspnea on exertion and a 2-pound weight gain. She denied nausea, vomiting, or abdominal distention. She was afebrile but tachycardic. Her lungs were clear on auscultation, abdomen was slightly distended with mild tenderness in the left lower quadrant. Ultrasound evaluation demonstrated no evidence of intraperitoneal fluid, and ovaries were enlarged bilaterally (left: 56 × 89 mm; right: 48 × 81 mm) consistent with moderate hyperstimulation. She had normal electrolytes and renal function, hematocrit was 0.44%; she was initially managed as an outpatient with oral hydration and modified activity. Her symptoms progressed such that by POD #12, she presented with a 5-pound weight gain and nonproductive cough. Her pulse was 118 bpm and saturating 97% on room air. On physical examination, breath sounds were absent in ~66% of the posterior right lung field. Her abdomen remained slightly distended, and on ultrasound, there was still no evidence of intraperitoneal fluid. A chest X-ray (CXR) confirmed a large right pleural effusion (Figure 1). Spiral CT scan was negative for pulmonary embolism (PE) or deep venous thrombosis (DVT) and confirmed a minimal amount of abdominal ascites. Thoracentesis was performed; 2600 cc clear yellow fluid was initially suctioned off with a total of 2900 cc over 48 hours. Total fluid protein was 37 g/L, which is consistent with exudates. Pleural fluid culture was negative. She was placed on low molecular weight heparin (LMWH) and sequential compression devices (SCDs) for DVT prophylaxis.

During her 2-day hospital admission, her dyspnea and nonproductive cough resolved. On day of discharge her CXR showed resolution of the pleural effusion, and, subsequently, the chest tube was removed without complications. She returned to our office 3 days after hospital discharge with a 9-pound weight loss and complete resolution of symptoms. The patient was followed closely as an outpatient with serial chest X-rays; she fully recovered without further sequelae.

### 1.2. Case  2

A 41-year-old G_3_P_1011_ woman with no significant past medical history presented for IVF-preimplantation genetic screening (PGS) for family balancing after conceiving her first child, without complications, with ovulation induction using gonadotropins and intrauterine insemination. Controlled ovarian hyperstimulation was performed using an antagonist protocol with daily injections of 300 IU of recombinant follicle-stimulating hormone (rFSH) and 150 IU of hMG. 250 *μ*g of Ganirelix Acetate injection was started on day 9 and continued for 4 days. 10,000 units of hCG intramuscularly were given when two lead follicles reached 18 mm. The patient received a total of 2550 IU of FSH and 1350 IU of hMG over 9 days. Her peak estradiol was 2552 pmol/L. 18 oocytes were retrieved. Two genetically normal embryos were transferred. Daily intramuscular progesterone was given for luteal phase supplementation.

Fourteen days after oocyte retrieval, the patient presented with complaints of nausea and bloating. Serum pregnancy test was positive. Her physical examination was significant for a 3-pound weight gain since the day of oocyte retrieval. She was tachycardic but saturating 98% on room air. Her abdomen was slightly distended and tender. Lung fields were clear to auscultation bilaterally. Ultrasound demonstrated minimal free intraperitoneal fluid, and ovaries measured 50 × 48 mm and 66 × 37 mm. Laboratory assessment was significant for normal electrolytes and renal function; hematocrit was 48.4%. The patient was initially managed conservatively as an outpatient with oral hydration and modified activity. However, less than 12 hours after her initial evaluation, the patient subsequently developed shortness of breath and chest pain and was admitted to the hospital. Her physical exam was significant for tachypnea and labored breathing with significantly decreased breath sounds from ~66% of the posterior right lung field. Repeat ultrasound evaluation demonstrated no evidence of free intraperitoneal fluid. CXR demonstrated a moderate right sided pleural effusion (Figure 2). Thoracentesis was performed; 2600 cc clear yellow fluid was initially suctioned off via pleurovac with a total drainage of 2750 cc over 3 days. Total fluid protein was 42 g/L, consistent with an exudative pleural effusion. Bacterial culture and fluid cytology were negative. She was placed on LMWH for DVT prophylaxis. The patient's course was complicated by several episodes of severe pleuritic chest pain requiring removal of chest tube on hospital day 2. A CT scan was performed which was negative for a PE. Her clinical symptoms resolved upon removal of the chest tube despite a residual small right pleural effusion on CXR. The patient was discharged home on hospital day 3 with a hematocrit of 41, which had decreased from a peak level of 48. Her serum sodium normalized at 136 meq/L after rising from a nadir of 130 meq/L. Serum beta hCG was 134 mIU/mL on day 28 and rose appropriately.

Two days after discharge, she began to experience worsening chest pain and shortness of breath. A repeat CXR demonstrated a large right pleural effusion which was significantly larger in comparison to prior CXR. The patient was readmitted to the hospital due to the severity of symptoms. A CT scan to rule out a PE and an echocardiogram to rule out underlying cardiac disease were both negative. The patient underwent a second thoracentesis with drainage of 2100 cc over the next 4 days. The pleural fluid analysis was consistent with an exudate and again demonstrated no malignant cells on cytology and negative bacterial culture. The patient experienced hyponatremia to a nadir of 130 meq/L which gradually resolved with intravenous normal saline treatment. On hospital day 5, the chest tube drainage was minimal (70 cc), and a chest X-ray demonstrated a significant improvement in pleural effusion. The chest tube was discontinued, and the patient was discharged home on hospital day 6.

The patient was followed as an outpatient with gradual resolution of pleuritic chest pain and no further complications. A twin intrauterine pregnancy was documented by ultrasound on cycle day 46. Despite one of the fetuses spontaneously miscarrying, the patient subsequently had an uncomplicated singleton pregnancy and delivered a healthy male infant at term.

## 2. Conclusion

OHSS usually results from stimulation of the ovaries by gonadotropins with the initial onset following the administration of exogenous hCG. Women at particularly higher risk include those with the following characteristics: younger age, polycystic ovaries, low BMI, rapidly rising serum estradiol level, and an elevated peak estradiol level [11]. OHSS has traditionally been classified as mild, moderate, or severe. Mild manifestations of OHSS are relatively common in ART and include transient lower abdominal discomfort, mild nausea, vomiting, diarrhea, and abdominal distention. Progression of the illness is recognized when symptoms persist, worsen, or include ascites that may be demonstrated by increasing abdominal girth or ultrasound evaluation. Symptoms generally resolve within 1 to 2 weeks but become more severe and persist longer if pregnancy is successful. 

Since the first case of isolated pleural effusion associated with severe OHSS was described in 1975, there have been few other published case reports [4, 5, 12–27]. We describe two unusual cases of OHSS where unilateral pleural effusion was the presenting symptom. Patient number 1's case is the first described case to present as early as 2 days after oocyte retrieval with symptoms of dyspnea. Prior to our case, the earliest described presentation was 6 days after oocyte retrieval [20]. Patient number 2's case is remarkable in that it is only the second case to be described in a woman over 40 years of age; the first was described in 2007 [21].

Complaints of dyspnea within the first few days after oocyte retrieval in the absence of intra-abdominal ascites should raise the physician's clinical suspicion of isolated pleural effusion. In these cases, a chest X-ray should be included in addition to the standard workup for OHSS. Physicians should not be misled in thinking that dyspnea is only a direct result of increased intra-abdominal pressure in the setting of OHSS, especially when ultrasound findings reveal low intra-abdominal fluid volumes. If a pleural effusion is diagnosed, then appropriate treatment may include drainage by thoracocentesis. This procedure was used safely in these two cases, which led to reversal of symptoms and an improvement in clinical status. 

In summary, as the number of IVF cycles performed increases, it is important to make physicians aware of this very real and perhaps underestimated complication of OHSS, as pleural effusions may significantly compromise the health of the patient if treatment is not initiated in a reasonable amount of time.

## Figures and Tables

**Figure 2:**